# Letter to the Editor: commentary on ‘Artificial intelligence-based diagnosis of standard endoscopic ultrasonography scanning sites in the biliopancreatic system: a multicenter retrospective study’

**DOI:** 10.1097/JS9.0000000000001559

**Published:** 2024-05-08

**Authors:** Shuang-Mei Dai, Tao Guo

**Affiliations:** Department of Gastroenterology, State Key Laboratory of Complex Severe and Rare Diseases, Peking Union Medical College Hospital, Peking Union Medical College and Chinese Academy of Medical Sciences, Beijing, People’s Republic of China

*Dear Editor,*

We read with interest the study on a convolutional neural network (CNN)-based model to automatically identify standard anatomical sites on endoscopic ultrasonography (EUS) images^1^. This paper conducted a multicenter retrospective study and proposed a new deep learning model that can help doctors identify and locate different structures in gastrointestinal endoscopy, explored the sensitivity, specificity, positive predictive value, and consistency rate of the model at different scanning locations. To some extent, this model may have the opportunity to assist doctors in identifying and locating the biliary and pancreatic systems more accurately and improving the accuracy and efficiency of diagnosis. This reveals that the application and development of machine learning in gastrointestinal endoscopy have great prospects.

Gastroenterology has been an early leader in bridging the gap between artificial intelligence (AI) model development and clinical trial verification^2–4^. This study has a wide application prospect; we very much appreciate the authors’ vision, but there are still some limitations. Firstly, the black-box nature of deep learning models makes their predictions difficult to interpret, which may also affect doctors’ trust and clinical application. Secondly, in this study, EUS images of standard anatomical sites are selected to train the CNN model; while human anatomy is variable, the accuracy of the CNN model in clinical application may not be as good as that of the trial. Then, while the model performed well in testing, taking into account differences in equipment and operators, the reliability and accuracy of the research results need to be further evaluated.

In short, in this era of booming artificial intelligence, we support the application of machine learning in EUS and believe that deep learning models can help doctors identify and locate different structures and even differentiate different diseases in gastrointestinal endoscopy. In the future, researchers can further expand the depth and breadth of the research and strengthen interdisciplinary integration in order to obtain more rich and comprehensive conclusions.

## Ethics approval

Not applicable.

## Consent

Not applicable.

## Sources of funding

This work was supported by the National Key Research and Development Program of the Ministry of Science and Technology, ‘Clinical Performance Verification and Case Collection of Electronic Loop Scanning Ultrasonic Endoscope’ [subject No.: 2017YFC0109804].

## Author contribution

T.G.: conceptualization, funding acquisition, methodology, supervision, and writing – review and editing; S.-M.D.: methodology and writing – original draft.

## Conflicts of interest disclosure

There are no conflicts of interest.

## Research registration unique identifying number (UIN)

Not applicable.

## Guarantor

Tao Guo and Shuang-Mei Dai.

## Data availability statement

Not applicable.

## Provenance and peer review

This paper was not invited.

## Disclaimer

The authors declare that the views expressed in the submitted articles are their own views, not the official position of the institution.

## Assistance with the study

Not applicable.
